# Efficacy evaluation of a commercially available MCT enriched therapeutic diet on dogs with idiopathic epilepsy treated with zonisamide: a prospective, randomized, double-blinded, placebo-controlled, crossover dietary preliminary study

**DOI:** 10.1186/s12917-023-03710-4

**Published:** 2023-09-06

**Authors:** Kazumasa Nakatsuka, Brian Zanghi, Daisuke Hasegawa

**Affiliations:** 1 Department of Veterinary Pharmacology, Faculty of Agriculture, Tottori University, Tottori, Japan; 2 Academic, Nestlé Purina PetCare, Kobe, Japan; 3 Global Nutrition and Communications, Nestlé Purina PetCare, St. Louis, USA; 4 Laboratory of Veterinary Radiology, Faculty of Veterinary Science, Nippon Veterinary and Life Science University, Tokyo, Japan; 5 The Research Center for Animal Life Sciences, Nippon Veterinary and Life Science University, Tokyo, Japan

**Keywords:** β-hydroxybutyric acid, Epilepsy, MCT oil, Medium-chain triglyceride, NeuroCare, Seizure, Zonisamide

## Abstract

**Background:**

Idiopathic epilepsy (IE) is a common, chronic brain dysfunction in dogs. Recently, the effect of feeding a diet enriched with medium-chain triglycerides (MCTs) on seizure frequency has been evaluated in several studies in dogs with IE. However, most dogs with IE in previous studies were treated with phenobarbital as the main antiseizure medication (ASM). In Japan, zonisamide (ZNS) is the most prescribed ASM for dogs with IE. The interaction between ZNS and various nutrients including MCTs and the potential effects on treatment efficacy resulting from combining these therapies have not been previously studied. A prospective, randomized, double-blinded, placebo-controlled, crossover dietary study was conducted. Dogs (*n* = 7) treated with ZNS were fed either a placebo diet (PL) or Purina ProPlan Veterinary Diet NeuroCare (NC) for 3 months, after which treatments were crossed over and continued for another 3 months. Seizure frequency (seizures/month; sz/m), blood tests including concentrations of ZNS and β-hydroxybutyric acid, and owner’s visual analogue scale score were collected from all dogs for both treatment periods.

**Results:**

There was no significant difference in the seizure frequency between PL (2.95 ± 0.80 sz/m) and NC (1.90 ± 0.57 sz/m) during the 6 months of trial. Three of 7 dogs showed ≥ 50% seizure reduction, and 1 of those 3 dogs achieved seizure freedom in NC period. However, 2 of 7 dogs had no changes in epileptic seizure frequency, 2 of 7 dogs had a deterioration in seizure frequency in the NC period. Feeding the MCT diet concurrent with ZNS showed no apparent adverse effects and did not affect ZNS concentration.

**Conclusions:**

This study indicated that the commercially available MCT-enriched diet (NC) can be safely used concurrently with ZNS for dogs with IE.

**Supplementary Information:**

The online version contains supplementary material available at 10.1186/s12917-023-03710-4.

## Background

Epilepsy is one of the most common, chronic neurological disorders in humans and dogs [1, 2]. The prevalence of epilepsy in dogs has been estimated at 0.6–0.75% in the general dog population and much higher in some pure breeds [3–6]. Idiopathic epilepsy (IE) is the most common etiology of epilepsy in dogs and is clinically defined as two or more unprovoked epileptic seizures at least 24 h apart with no identifiable underlying etiology other than a suspected genetic origin [3].

Antiseizure medication (ASM) regimen is the main treatment used to control epileptic seizures (ES). Phenobarbital (PB) and zonisamide (ZNS) are commonly used ASMs as the first-line treatment in Japan [7]. Because ZNS is not available in every country, there have been few reports regarding the efficacy and adverse effects in the prevention of ES in canine IE [8, 9]. Although the exact mode of action underlying the antiseizure effect of ZNS are still unclear, it is thought to block both T-type calcium and voltage-gated sodium channels, enhance GABA release, and inhibit glutamate release as contributing factors influencing the antiseizure effects of this drug [10]. The commonly observed adverse effects of ZNS are sedation, vomiting, ataxia, and decreasing appetite. The decrease in appetite sometimes associated with ZNS contrasts significantly with the polyphagia that is commonly associated with other ASMs including PB, potassium bromide, and imepitoin [11, 12].

In human epilepsy studies, the response rate to first-line, second-line, and third-line ASMs have been investigated, and each response rate is 47%, 13%, and 4%, respectively [13]. Similar results have been reported in dogs with IE with response rates reported at 37%, 11%, and 6%, respectively [14]. Notably, when the patients did not respond to first-line ASMs, the chances of responding to second- and third-line ASMs are progressively lower [14]. Therefore, additional therapies for patients with ASM-resistant epilepsy are desired. Although epilepsy surgery has been established for ASM-resistant epilepsy in human medicine [15, 16], surgery to address canine epilepsy is generally limited to experimental studies or a few case reports in veterinary medicine [17–22].

Ketogenic diet therapy is an additional option for people with ASM-resistant epilepsy. Ketogenic diets were introduced for childhood epilepsy in the 1920s and consisted of high fat and low carbohydrate intake with a ratio up to 4:1 for fat to carbohydrate and protein [23]. An initial diet intervention study using a classic ketogenic diet was performed to assess the efficacy in dogs with epilepsy but showed no significant difference in ES frequency [24].

As an alternative to a classic ketogenic diet, medium-chain fatty acids (MCFAs) have been reported to exert antiseizure effects in a dose-dependent manner [25]. MCFAs can be bioavailable following the ingestion, digestion, and metabolism of medium-chain triglycerides (MCT) composed of a glycerol backbone and three MCFAs with the aliphatic tail of 6–12 carbon atoms. MCTs exist in some natural foods such as coconut oil and palm kernel oil according to the US Department of Agriculture National Nutrient Database [26]. In a recent study, MCT-enriched diets (MCTD) were fed to dogs with ASM-resistant IE [23]. The study demonstrated that 14% (3/21 dogs) achieved ES freedom, 48% (10/21 dogs) had ≥ 50% reduction in ES frequency, 24% (5/21 dogs) had no change, and 29% (6/21 dogs) had an increase of ES frequency in MCTD period compared to Placebo (PL) period. This study also reported that 14% (3/21 dogs) achieved ES freedom, 33% (7/21 dogs) had ≥ 50% reduction in ES day frequency, 48% (10/21 dogs) had no change, and 19% (4/21 dogs) had an increase of ES day frequency in MCTD period vs PL period. A subsequent canine study resulted in similar observations when the test diet contained 6.5% MCT oil, high levels omega-3 fatty acids, and other nutrient enrichments: 2/21 (9.5%) dogs became seizure free, 9/21 dogs (42.9%) had ≥ 50% reduction, 7/21 (33.3%) dogs had < 50% reduction, 1/21 (4.8%) had no change and 4/21 dogs (19.0%) had an increase in ES frequency [27].

In both studies, nearly all dogs were treated with PB as the main ASM and none included ZNS. Therefore, no evidence exists to demonstrate whether MCTD is also effective as an adjunct for dogs with ASM-resistant IE and treated with ZNS, as there is currently no published information about the combined use of MCTD and ZNS for dogs with IE.

The objective of this study was to investigate the efficacy of a commercially available therapeutic diet enriched with 6.5% MCT oil and other nutrients such as omega-3 fatty acids, arginine, and vitamin E (NC; Purina Pro Plan NC NeuroCare, Nestlé Purina, St. Louis Missouri, USA) in dogs with ASM-resistant IE using ZNS as the main ASM. According to Japan Veterinary Medical Association, the therapeutic diet is defined as a pet food with special product characteristics that differ from general health maintenance diets due to special nutritional characteristics and special manufacturing methods, etc. The therapeutic diet is also adjusted with the intention of using for dietary therapy and is regulated by several laws in Japan [28]. NC is MCT oil enriched diet, and the medical indications are IE and cognitive dysfunction syndrome [29]. We hypothesized that daily ingestion of NC will have similar efficacy in IE dogs treated with ZNS as the effect observed in IE dogs treated with PB (± bromide) in previous studies. In order to verify our hypothesis and confirm the safety of the combination of MCTD (NC) and ZNS, we conducted a prospective, randomized, double-blinded, placebo-controlled, crossover dietary study in dogs with IE treated with ZNS.

## Results

### Study participants

Sixteen dogs were screened for recruitment from 15 veterinary clinics, but 8 dogs did not meet the inclusion criteria. Eight dogs were recruited to start the study, but data from 1 dog was excluded when it was determined that the ASM dose was changed during the study, thus only 7 dogs (Dogs 1–7) completed this study. Participant signalment including breed, age, sex, ES frequency, cluster seizure presence/absence, diet in 3 months pre-trial period, body weight (BW), body condition score (BCS) at entry, and using ASMs are shown in Table 1. The dogs participating in the trial included one of each of the following breeds: Australian Shepherd (Dog 1), Shetland Sheepdog (Dog 2), Chihuahua (Dog 3), Toy poodle (Dog 4), Cavalier King Charles Spaniel (Dog 5), French Bulldog (Dog 6), and Siberian Husky (Dog 7). The study participants consisted of four males and three females. All females were spayed, and two of four males were neutered. Age, BW, and BCS of participants at the entry of the trial were 6.14 ± 2.90 years old, 11.55 ± 8.19 kg, and 4.71 ± 0.45 BCS, respectively. All participants were prescribed ZNS (Dogs 1–7, 7.01 ± 2.31 mg/kg, BID) as the main ASM with three dogs also prescribed additional ASMs (Dog 1, potassium bromide and pregabalin; Dog 5, pregabalin; Dog 7, gabapentin). There were no significant differences in BW and BCS between the end of placebo (PL) period and the end of NC period.

**Table 1:** Dogs breed, age, sex, epileptic seizure frequency, presence/absence of cluster seizures, diet in 3 months pre-trial period, body weight, body condition score, and antiseizure medications

Dog #	Breed	Age at entry (y)	Sex	ES frequency in 3 months pre-trial period (sz/m)	Cluster seizure presence / absence in 3 months pre-trial period	Status epileptics presence / absence in 3 months pre-trial period	Diet in pre pre-trial period	BW at entry (kg)	BW end point of PL (kg)	BW end point of NC (kg)	BCS at entry	BCS end point of PL	BCS end point of NC	ASMs
Dog 1	Australian Shepherd	5	NM	6	Presence	Absence	Complete balanced diet	20.5	23.0	23.6	5	6	7	ZNS, PGB, KBr
Dog 2	Shetland Sheepdog	6	NM	3	Presence	Absence	Urinary therapeutic diet	11.4	12.8	12.3	5	5	5	ZNS
Dog 3	Chihuahua	9	M	6	Presence	Absence	Complete balanced diet	3.4	3.0	3.3	4	4	4	ZNS
Dog 4	Toy poodle	10	NF	4	Absence	Absence	Complete balanced diet	1.7	2.0	1.8	5	5	5	ZNS
Dog 5	Cavalier KC Spaniel	4	NF	5	Absence	Absence	Urinary therapeutic diet	7.6	7.6	7.7	5	5	5	ZNS, PGB
Dog 6	French bulldog	1	M	3	Presence	Absence	Complete balanced diet	10.3	10.9	10.8	4	4	4	ZNS
Dog 7	Siberian husky	8	NF	20	Presence	Absence	Complete balanced diet	26.0	26.1	28.0	5	5	5	ZNS, GBP
Mean ± S.D		6.14 ± 2.90						11.55 ± 8.19	12.19 ± 8.64	12.49 ± 9.16	4.71 ± 0.45	4.85 ± 0.64	5.00 ± 0.93	

*ES *epileptic seizure*, BW* Body weight, *BCS* Body condition score, *PL* Placebo diet, *NC* NeuroCare diet, *ASMs* Antiseizure medications, *NM* Neutered male, *M* Male, *NF* Neutered female, *F* Female, *ZNS* Zonisamide, *PGB* Pregabalin, *KBr* Potassium bromide, *GBP* Gabapentin

### Epileptic seizure frequency and epileptic seizure days frequency

**Fig. 1:**
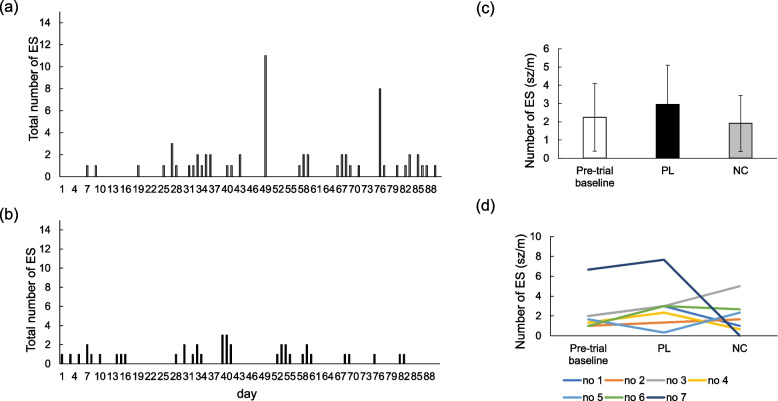
Effect of the placebo (PL) on epileptic seizure (ES) frequency in 3 months (**a**) and effect of NeuroCare (NC) on ES frequency in 3 months (**b**) (*n* = 7). There was no significant difference in the number of ESs per month between PL and NC (**c**, *p* = 0.348). Changes in each dog's ES frequency per month in pre-trial, PL, and NC periods (**d**)

The distribution of all ES events in each 3-month experimental period is illustrated graphically for the PL (Fig. 1a) and NC (Fig. 1b) treatment groups. Although the ES frequency did not differ (*P* = 0.37) between NC and PL periods, there was a numerical decline of 35% in the ES frequency per month (sz/m) for dogs ingesting the NC diet (1.90 ± 1.53 sz/m) compared to the PL diet (2.95 ± 2.14 sz/m; Fig. 1c) The ES frequency between pre-trial period (2.23 ± 1.84 sz/m; Fig. 1c) and NC period also did not differ (*P* = 0.74). Only 3 of 7 dogs experienced partial treatment success according to the IVETF definitions [8]. From an observational perspective, one dog (Dog 7; 14%) achieved seizure freedom during the 90-day NC period compared to the PL period; this dog experienced 2 cluster seizures events on separate days (day 49 and day 76, 11 ESs and 8 ESs per day for each) during the PL period with 6 total ES days recorded and also had more than 20 cluster seizure events over 5 days in 3 months pre-trial period. Four dogs (Dogs 1, 2, 3, and 6) also had 1 cluster seizure event with 2 ES during pre-trial period. While eating the NC diet, 3 dogs (Dogs 1, 4, and 7; 42%) were observed to have ≥ 50% reduction in ES frequency, 2 of 7 dogs (Dogs 2 and 6; 29%) experienced no change in ES frequency (± 25% difference from PL), and 2 of 7 (Dogs 3 and 5; 29%) had an increase in ES frequency compared to PL diet. Each dog's ES frequency per month in pre-trial period, PL period, and NC period were shown in Fig. 1d.

**Fig. 2:**
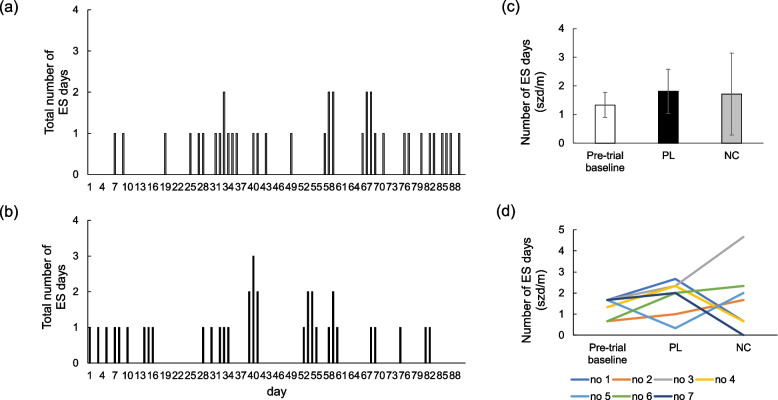
Effect of the placebo (PL) on epileptic seizure (ES) days in 3 months (**a**) and effect of NeuroCare (NC) on ES days in 3 months (**b**) (*n* = 7). There was no significant difference between the number of ES days per month between PL and NC (**c**, *p* = 0.888). Changes in each dog's ES days per month in pre-trial, PL period and NC period (**d**)

The distribution of all ES days in each 3-month experimental period is illustrated graphically for the PL (Fig. 2a) and NC (Fig. 2b) treatment groups. Similar to ES frequency above, the ES days per month did not differ (*P* = 0.89) between NC and PL periods with only a 6% numerical decline in number of ES days (szd/m) when dogs were on the NC period (1.71 ± 1.43 szd/m) compared to the PL period (1.81 ± 0.77 szd/m; Fig. 2c). ES days per month also did not differ (*P* = 0.54) between NC period and pre-trial period (1.33 ± 0.44). Each dog ES days per month in pre-trial period, PL period, and NC period were showed in Fig. 2d.

To further evaluate the data, a post-hoc analysis of the data was conducted to assess a month-by-month change in ES frequency and ES days per month through calculating ES data separately for each month of the experimental periods. Because of the limited number of pets in the study, the dataset lacked the necessary degrees of freedom to conduct appropriate statistical analysis and most statistical models were unable to converge the results. In the NC period, 3 dogs were seizure free and 1 dog had 2 ESs only. By contrast in the PL period, 1 dog was seizure free and 1 dog had a single ES, whereas the other 5 dogs had between 3 to 10 ESs recorded.

### Visual analogue scale score and adverse events

**Fig. 3:**
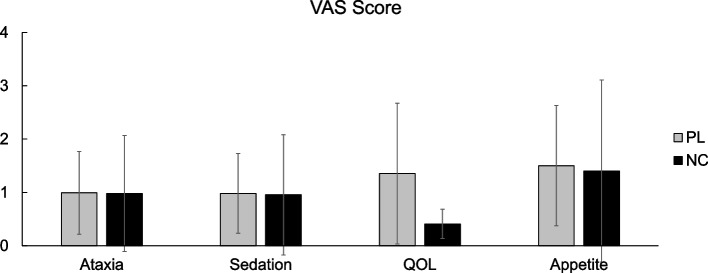
Visual analogue scale (VAS) score for ataxia, sedation, appetite, and QOL at the endpoint of placebo (PL) and at the end of NeuroCare (NC). There was no significant difference for all parameters compared at the end of PL with at the end of NC

Visual analogue scale (VAS) score (mean and S.D.) for each evaluated item for PL and NC groups are shown in Fig. 3. For each item, there were no significant differences between the treatment groups (*P*-values were 0.982, 0.964, 0.907, and 0.112, for ataxia, sedation, appetite, and QOL, respectively), although the mean QOL score seemed to be improved.

No adverse event was reported from dogs no. 1, 3, and 5. Dog no. 2 showed soft feces for three days while in the NC period. Dog no. 4 showed decreased appetite, diarrhea, and vomiting for several days in both PL and NC periods. Dog no. 6 showed soft feces and decreased appetite for two days in the NC period, and also showed diarrhea, soft feces, and hematuria for one day in the PL period. Dog no.7 showed nausea for one day in the NC period.

### Concentration of plasma ZNS, serum ketone bodies, CBC, and serum chemistry

Results of plasma ZNS and serum ketone concentrations and other blood results are shown in Table 2 and Supplementary file 1, respectively. No significant difference was observed in plasma concentration of ZNS at the end of PL period (19.70 ± 6.78 µg/ml) compared to the end of NC period (20.89 ± 10.68, µg/ml; *P* = 0.822), serum total ketone concentration (PL; 37.86 ± 11.29 µmol/l vs. NC; 43.86 ± 16.13 µmol/l; *P* = 0.102), serum acetoacetic acid (PL; 10.41 ± 5.08 µmol/l vs. NC; 10.43 ± 4.24 µmol/l; *P* = 0.993) or for any CBC and serum chemistry measure (Supplementary file 1). Serum β-hydroxybutyric acid level was significantly different (*P* = 0.048) at the end of PL period (22.65 ± 4.24 µmol/l) compared to the end of NC period (34.14 ± 12.08 µmol/l) (Table 2).

**Table 2:** Concentrations of zonisamide and blood ketone levels at each time point

	Entry		Start point of PL		End point of PL		Start point of NC		End point of NC	
	Average	S.D	Average	S.D	Average	S.D	Average	S.D	Average	S.D
ZNS (μg/mL)	23.24	6.65	20.89	10.08	19.7	6.78	18.19	11.78	20.89	10.68
Total ketone body (μmol/L)	30.29	22.4	35.29	14.56	37.86	11.29	37.71	14.64	43.86	16.13
Acetoacetic acid (μmol/L)	6.43	3.81	14.86	15.68	10.41	5.08	14.29	17.19	10.43	4.24
β-hydroxybutyric acid (μmol/L)	23.86	18.88	20.66	3.76	22.65	4.24	23.43	6.86	34.14 *	12.08

*PL* Placebo diet, *NC* NeuroCare diet, *S.D* Standard deviation, *ZNS* Zonisamide. Generalized linear mixed model with diet as fixed effect and dog as random effect was used to compare

^*^P < 0.05

## Discussion

This study was performed to assess the influence of feeding a commercially available therapeutic MCTD (NC) for dogs with ASM-resistant IE treated with ZNS as a main ASM. Although the study resulted in no statistical difference between the PL or NC diet on ES frequency or ES days, some observational assessment of the individual data suggests that some ZNS-treated dogs with IE ingesting the NC diet may have some decrease in ES frequency and fewer ES days, especially in the last 30 days of a 3-month period (Figs. 1a, b and 2a, b). Conversely, 2 of 7 dogs showed an increase in ES frequency, which was similar to previous study reports [23]. A significant limitation of this study is the small number of recruited pets, with only 7 cases compared to the previously published studies [23, 30], and thus is significantly underpowered. This study does provide some important and relevant observations of dogs ingesting the NC diet. Several measures did not change over the feeding period, specifically ZNS concentration, BW, ataxia, sedation, appetite, and blood profiles. Although there was no statistically significant, some owners reported an improvement in the patient’s QOL while ingesting the NC (this is also discussed later).

A previously published study evaluating the feeding of an MCTD to dogs with IE and treated with PB reported a significant reduction of ES frequency over 90 days. More specifically, ES frequency was reduced in 71% dogs, > 50% seizure reduction in 48% dogs, and seizure freedom in 14% dogs [23]. Similar results were reported from an open-label feeding trial using NC same as the present study [27] and an MCT supplement trial [30]. In the present study using ZNS as the main ASM, 42% dogs showed ≥ 50% ES frequency reduction, with 1 dog (14%) becoming seizure free. On a relative basis, these values are very similar to previous study results [23, 29, 30]. In the present study, 5 dogs during the PL period experienced cluster seizures with a total of 8 cluster seizure events, whereas 4 dogs during the NC period each had a single cluster event of only 2 ESs.

There was a significant increase in serum β-hydroxybutyric acid concentration at the end of the NC period compared to the end of PL period. MCTs are metabolized into MCFAs which can quickly be metabolized into ketone bodies such as β-hydroxybutyric acid [31]. There are some reports that epilepsy is related to impairments in brain glucose metabolism in humans, rodent epilepsy models, and dogs [32–35]. β-hydroxybutyric acid might provide an alternative energy source in such status and contribute to seizure reduction [36, 37]. MCFAs such as octanoic acid and decanoic acid have been reported to exert an antiseizure effect via α-amino-3-hydroxy-5-methyl-4-isoxazole propionic acid (AMPA) receptor inhibition [26], and this might also be a reason for seizure reduction. Perampanel, a relatively new ASM for human epilepsy, is a non-competitive antagonist of AMPA receptor, and the site of action is similar to MCFAs [38–40]. In the rat amygdaloid kindling model of temporal lobe epilepsy, a clear antiseizure effect and the binding site of perampanel are revealed [41]. Based on the above, we expected that the MCTs in NC would be degraded into MCFAs and would confirm the antiseizure effect. However, we could not reach a clear conclusion due to the small number of animals as described in the limitation.

In the present study, there were no significant differences in VAS scores by owners, however, patients’ QOL tended to improve in the NC period (*p* = 0.11). Owners of dogs who achieved the greatest quantitative seizure reduction also generally reported a good impression of the diet on the pet. Owners of animals with chronic disease(s) may experience signs of stress, anxiety, and depression due to the burden of care [42]. In canine epilepsy, ESs are difficult to predict, and the anxiety of not knowing when they will occur is always with the owners. A frequency of less than one ES every three months is associated with the owners’ perception of adequate ES control [43]. Therefore, the ES frequency reduction may have impacted the QOL assessment of the VAS score. Appetite is also a key factor for the success of dietary medication. PB has a well-known side effect of hyperphagia, while ZNS use results in a decreasing appetite as the main side effect. However, there was no change in both appetites on the VAS score and BW in the present study. This study demonstrates that the NC is well tolerated in dogs with IE treated with ZNS.

A critical limitation of this study is the small number of recruited dogs, which in part was impacted by the COVID-19 pandemic, which unfortunately negatively influenced the ability to adequately promote the trial and recruit sufficient pets from local veterinary clinics. Ultimately, only 7 dogs completed this trial, which is considerably fewer than other published studies that demonstrated a significant dietary influence to reduce ES frequency. Although the results of this study do not demonstrate a clinical difference between the diet groups, the NC diet can be safely used concurrently with ZNS for dogs with ASM-resistant idiopathic epilepsy. It is possible that long-term feeding will be required to realize a beneficial effect for the pet. A larger-scale and longer duration treatment period may reveal more evidence of interactions between MCT and ZNS.

## Conclusion

This is the first study to identify the effect of MCT enriched therapeutic diet (NC) for dogs with IE using ZNS as first line ASM. Although there is no statistically significant in ES frequency during the 3-month study period, it is a safety treatment option for dogs with ASM-resistant idiopathic epilepsy.

## Materials and methods

### Study design and ethics

A prospective, randomized, double-blinded, placebo-controlled, crossover dietary protocol was designed with pet recruitment to occur as a multi-institutional study within Japan. The study protocol was approved by the Veterinary Medical Teaching Hospital of Nippon Veterinary and Life Science University (approved #: H30-2). All participant dogs were client-owned and enrolled only after the owner’s consent was obtained. During all study periods, the dose of ASM(s) was not changed, and the dogs were not fed anything (including treats) other than study diets. A commercially available diet (Purina Pro Plan NC NeuroCare, Nestlé Purina, St. Louis Missouri, USA) was used as the test diet (NC), whereas the placebo diet (PL) excluded MCT oil (substituted with beef fat), excluded omega-3 fatty acid, and contained lower levels of vitamin E and arginine. All other nutrients and caloric content of the PL were similar to NC. Proximate analyses and nutrient results of diets are shown in Table 3.

**Table 3:** Nutrient content and ingredient composition on dry matter basis of placebo and NeuroCare diets

Proximate analyses	PL	NC
Moisture (%)	7.1	8.4
Crude protein (%)	32.7	32.8
Crude fat (%)	19.4	17.6
Carbohydrate (%)	38.7	40.7
Crude fiber (%)	1.9	1.8
Ash (%)	7.3	7.2
Chloride (%)	0.7	1
Vitamin E (IU/kg)	907	1377
EPA + DHA (%)	0.02	0.52
Selenium (%)	0.7	0.7
Arginine (%)	1.9	2.7
MCT oil (%)	0	6.5
Energy value (kcal/100 g)	385	372

Proximate analysis. *PL* Placebo, *NC* NeuroCare

**Fig. 4:**
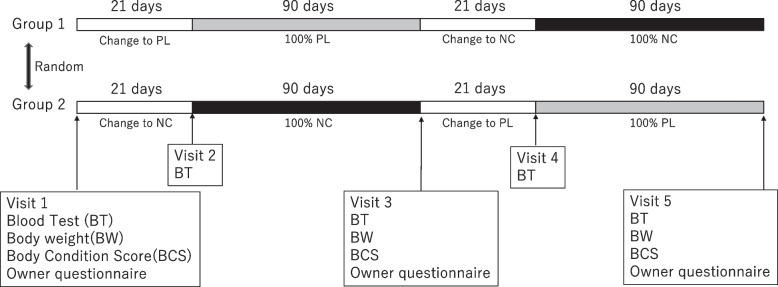
Study designed as 6-month prospective, randomized, double-blinded, placebo-controlled, cross-over dietary trial. Group 1 contained 4 dogs and Group 2 contained 3 dogs. The protocol of Group 1 consisted of 4 periods. The 1st period was 21 days for transitioning to Placebo (PL). The 2nd period was 90 days using PL only. The 3rd 21 days period, was a wash-out, and transitioning diets from PL to NeuroCare (NC). The 4th period was 90 days using NC. Group 2 feeding protocol was switched

The total study duration per pet subject was planned for 222 days. Subjects were diet-transitioned for 21 days to either the NC or PL diet to allow for 100% ingestion of the diet for the initial 3-month feeding period (Day 22 to Day 111 ± 2 days). This was followed by a subsequent respective switch of diet for a 21-day cross-over diet transition period (Days 112 to 132) before beginning the second 3-month treatment period (Day 133 to Day 222 ± 2 days). Protocol timeline and sample collection days are illustrated in Fig. 4. Samples and data were collected at visit 1 (Day 1), visit 3 (Day 111 ± 2 days) and visit 5 (day 222 ± 2 days), which included BW, BCS, blood tests for CBC and serum chemistry profile, and an owner-completed questionnaire with VAS described below. Only blood samples were also collected at visit 2 (day 21) and visit 4 (day 133).

In this study, cluster seizure was defined as two or more seizures within a 24-h period according to the International Veterinary Epilepsy Task Force (IVETF)’s consensus report [6]. And also, we defined the treatment success by a ≥ 50% reduction in ES frequency of both NC and PL periods compared to the pre-trial period according to the IVETF’s consensus proposal [44].

### Participant recruitment and inclusion criteria

As the inclusion criteria for this trial, all participant dogs had to be previously diagnosed with IE by the International Veterinary Epilepsy Task Force’s tier I confidence level [45] and had to have more than three generalized tonic–clonic ESs (regardless of primary or evolving from focal ESs) in the last 3 months before inclusion. They had to be prescribed ZNS as the main ASM for more than half a year before inclusion. The age at participation was between 1 and 10 years. There was no restriction on BW for inclusion, as well as no clinically significant findings on CBC, serum chemistry, and dynamic bile acid results. Dogs who had other identifiable diseases, were pregnant, or were lactating were excluded. If the veterinarian and owner considered it was not appropriate to continue the clinical trial due to the patient’s condition, they could withdraw from the trial at any time.

### Blood samples

During the study, all blood samples from participants were collected 2 h after consumption of each diet and routine concomitant ASMs. Blood tests were performed at visits 1–5. CBC was measured in each clinic. The serum concentration of ketones (total ketones, acetoacetic acid, and ß-hydroxybutyric acid), the plasma concentration of ZNS, and serum chemistries were measured at an external laboratory (Marupi Lifetec Co., Ltd., Japan). All serum and plasma samples were frozen immediately after the collection and sent to the external laboratory.

### Visual analogue scale score and adverse events

Visual analogue scale (VAS) scores using a 0–100 mm range for each scale line were measured to investigate the severity of sedation, ataxia, appetite, and patient’s quality of life (QOL). The VAS sheet used in this study (Supplementary file 2) was based on the previous study [23], but ‘appetite’ was added as the assessment item for this study. Owners drew an intersecting line on the 100 mm scale line which represented each subject severity based on the owner’s impression. The 0–10 mm represents normal, and a maximum of 100 mm, i.e., extreme VAS scores were interpreted as deep sleep, unable to walk, never eat diets, and deterioration that could result in euthanasia were associated with sedation, ataxia, appetite, and patient’s QOL, respectively. When adverse events occurred, owners recorded the date and their condition.

### Statistical analysis

ES frequency refers to the number of ESs per month (sz/m), and ES day frequency refers to the number of days per month with ES occurrence (szd/m). Diet treatment effect (NC vs PL) of ES frequency, ES day frequency, blood tests, and VAS score were analyzed using the generalized linear mixed model with diet as fixed effect and dog as random effect. BW and BCS among the entry period, after PL and NC, one-way ANOVA followed by the Turkey-Kramer test was used. All numerical data were shown as mean and standard deviation (S.D.). Treatment effects were considered statistically different using an alpha = 0.05.

### Supplementary Information

**Additional file 1: Supplementary file 1.** Complete blood count and clinical chemistry at each time point in two groups.

**Additional file 2:** **Supplementary file 2.** Sample of the visual analogue scale (VAS) sheet used in this study.

## Data Availability

The datasets used and/or analysed during the current study are available from the corresponding author on reasonable request.

## Abbreviations

ASM: Anti-seizure medication
BCS: Body condition score
BW: Body weight
CBC: Complete bland count
ES: Epileptic seizure
IE: Idiopathic epilepsy
MCFAs: Medium-chain fatty acids
MCT: Medium-chain triglyceride
MCTD: MCT diet
NC: NeuroCare
PB: Phenobarbital
PL: Placebo diet
QOL: Quality of life
VAS: Visual analogue scale
ZNS: Zonisamide
