# Supplementary material for: Efficacy evaluation of a commercially available MCT enriched therapeutic diet on dogs with idiopathic epilepsy treated with zonisamide: a prospective, randomized, double-blinded, placebo-controlled, crossover dietary preliminary study
Source: BMC Vet Res. 2023 Sep 6;19:145. doi: 10.1186/s12917-023-03710-4 (PMC10481612; doi:10.1186/s12917-023-03710-4)
Supplement: Supplementary file 2 — Additional file 2: Supplementary file 2. Sample of the visual analogue scale (VAS) sheet used in this study. [file 12917_2023_3710_MOESM2_ESM.pdf]

Supplementary file 2. Sample of the visual analogue scale (VAS) sheet used in this study.

VAS form Day0

ATAXIA

normal	ATAXIA VAS Assessment	Ataxia as severe that animal cannot walk

Measurement (mm)	

Sedation

normal	Sedation VAS Assessment	Animal only sleep

Measurement (mm)	

Quality of life

normal	QOL VAS Assessment	I would request euthanasia because the QOL of my dog is poor

Measurement (mm)	

Appetite

normal	Appetite VAS Assessment	Animal do not eat anything

Measurement (mm)	

Name of dog ______________

date (dd-mmm-yy)
